# Characterization of a Deep-Sea Actinobacterium Strain Uncovers Its Prominent Capability of Utilizing Taurine and Polyvinyl Alcohol

**DOI:** 10.3389/fmicb.2022.868728

**Published:** 2022-05-23

**Authors:** Yingqi Tan, Yeqi Shan, Rikuan Zheng, Rui Liu, Chaomin Sun

**Affiliations:** ^1^Chinese Academy of Sciences and Shandong Province Key Laboratory of Experimental Marine Biology and Center of Deep Sea Research, Institute of Oceanology, Chinese Academy of Sciences, Qingdao, China; ^2^Laboratory for Marine Biology and Biotechnology, Qingdao National Laboratory for Marine Science and Technology, Qingdao, China; ^3^College of Earth Science, University of Chinese Academy of Sciences, Beijing, China; ^4^Center of Ocean Mega-Science, Chinese Academy of Sciences, Qingdao, China

**Keywords:** actinomyces, deep sea, cultivation, taurine, polyvinyl alcohol

## Abstract

*Actinobacteria* represent a large group of important prokaryotes with great application potentials and widely distribute in diverse natural environments including the ocean. However, compared to their terrestrial cultured members, there are much less available marine *Actinobacteria*, especially deep-sea counterparts. Here, we cultured a bacterial strain of deep-sea actinobacterium, *Marmoricola* sp. TYQ2, by using a basal medium supplemented with taurine. Consistently, the growth of strain TYQ2 was significantly promoted by the supplement of taurine. Transcriptomic analysis showed that the expressions of genes encoding proteins associated with taurine metabolization and utilization as well as energy generation were evidently up-regulated when taurine was added. Moreover, strain TYQ2 was demonstrated to degrade polyvinyl alcohol (PVA) with the involvement of the redox cycle of extracellular quinol and quinone and the reduction of iron to ferrous, and strain TYQ2 could utilize the degradation products for energy production, thereby supporting bacterial growth. Overall, our experimental results demonstrate the prominent degradation capabilities of *Marmoricola* sp. TYQ2 toward the organics taurine and PVA.

## Introduction

Microorganisms play a vital role toward the decomposition of organic matter in the marine environment and the biogeological cycle (Abirami et al., 2021). Among these microbes, the microbial community in the bottom sediments of the deep sea formed an unexplored biosphere (Baker et al., 2021). Due to the difficulty of sampling and the complexity of the community structure, current researches lacked a full understanding of its unique biological and metabolic characteristics. However, it is sure that these microorganisms play crucial roles toward the global cycle of elements and nutrients in the deep biosphere (Zhang et al., 2020; Baker et al., 2021; Suzuki et al., 2021; Zheng et al., 2021).

Among the marine microorganisms, *Actinobacteria* members are considered as key members due to their wide distribution and diverse biological functions (Rathore et al., 2021). The *Actinobacteria* bacteria ubiquitously distribute in marine sediments (Solano et al., 2009), sea water (Sheikh et al., 2019), marine organic aggregates (Lam, 2006), marine sponges (Gandhimathi et al., 2009; Sheikh et al., 2019), and deep-sea gas hydrate reservoirs (Wang et al., 2014). Notably, *Actinobacteria* have broad application potentials, such as production of large number of secondary metabolites for developing novel antibiotics (Wang et al., 2014; Kamjam et al., 2017); production of variety of enzymes including alkaline protease, xylanase, α-galactosidase (Temuujin et al., 2016; Sanjivkumar et al., 2017; Thakrar and Singh, 2019). In addition, *Actinobacteria* could degrade and metabolize foreign compounds such as heavy metals, hydrocarbons, pesticides and plastics, and they are also potential candidates for bioremediation (Rathore et al., 2021).

*Nocardioidaceae* members are heterotrophic aerobic bacteria belonging to the phylum *Actinobacteria*. Like other *Actinobacteria*, they are considered to be consumers of organic substances in the ecosystem. Many biological groups in this family could metabolize refractory and foreign compounds by secreting a series of extracellular enzymes (Evtushenko and Ariskina, 2015). They may also use organic matter or the input of some atmospheric gases and minerals to participate in various chemical energy and nutrient metabolic processes (Evtushenko and Ariskina, 2015).

The discovery of novel actinomycete strain with unique metabolic activity from deep-sea samples clearly illustrate that indigenous deep-sea *Actinobacteria* indeed exist in the oceans and are important sources of novel secondary metabolites, exogenous substance degradation and biosurfactant production (Rathore et al., 2021; Stubbins et al., 2021). The study of *Actinobacteria* has become one of the hot spots of current research. However, the isolation and culture of deep-sea *Actinobacteria* are still facing technological barriers associated with isolation strategies. It is crucial to cultivate novel deep-sea *Actinobacteria* with innovative approaches. In the present study, we successfully cultivated an actinomycete strain *Marmoricola* sp. TYQ2 from the deep-sea cold seep by using a medium supplemented with taurine as the only carbon source. The novel strain belonged to the *Nocardiaceae* family. Combining the physiological, genomic, and transcriptomic methods, we specifically disclosed the metabolic pathways and energy production of taurine and polyvinyl alcohol (PVA) mediated by strain TYQ2 and corresponding contributions to its growth. The utilization of taurine and PVA by strain TYQ2 was demonstrated.

## Materials and Methods

### Sampling, Cultivation Conditions, and Strain Isolation

The sediment and water samples were collected by *RV KEXUE* from a deep-sea cold seep in the South China Sea in 2020. The sediment samples were diluted by the sterile seawater and spread on the solid basal medium supplemented with taurine (1.25 g/L). The components of basal medium were 60 g NaCl, 8.36 g MgCl_2_⋅6H_2_O, 6.8 g MgSO_4_⋅7H_2_O, 0.66 g KCl, 0.5 g NH_4_Cl, 0.212 g CaCl_2_, 15 g agar in 1 liter distilled water, pH 7.5. After autoclaving, the other components, including 4 mL/L 7.5% NaHCO_3_, 1 mL/L phosphate solution (K_2_HPO_4_, 140 g/L), 1 mL/L vitamin solution (5 mg/L p-aminobenzoic acid, 2 mg/L biotin, 10 mg/L pyridoxine hydrochloride, 5 mg/L thiamine hydrochloride, 5 mg/L Ca-pantothenate, 0.1 mg/L cobalamin, 2 mg/L folic acid, 5 mg/L riboflavin, 5 mg/L niacin, 5 mg/L lipoic acid), 25 mL/L taurine solution (50 g/L), 1 mL/L trace element solution (2.1 g/L FeSO_4_⋅7H_2_O, 30 mg/L H_3_BO_3_, 100 mg/L MnCl_2_⋅4H_2_O, 190 mg/L CoCl_2_⋅6H_2_O, 24 mg/L NiCl_2_⋅6H_2_O, 2 mg/L CuCl_2_⋅2H_2_O, 144 mg/L ZnSO_4_⋅7H_2_O, 36 mg/L Na_2_MoO_4_⋅2H_2_O, 5.2 g/L Na_2_EDTA⋅2H_2_O), were added and sterilized by filtration and stored at −20°C. In addition, to prevent fungal growth, 30 mg/L nystatin was added to the medium. Single bacterial colonies from the original medium were picked and purified. The purified strain was stored at −80°C using 1/10 2216E medium (0.5 g peptone and 0.1 g yeast extract in per liter of seawater) supplemented with 10 mM taurine and 20% (v/v) glycerol. The isolation and cultivation process and subsequent experiments were all conducted at normal atmospheric pressure and temperature.

### Transmission Electron Microscopy Observation

To obtain the cellular morphological characteristics of strain TYQ2, its cell pellets were collected for observation using TEM with a JEOL JEM 12000 EX (HT7700, Hitachi, Tokyo, Japan) equipped with a field emission gun at 100 kV. Briefly, the bacteria solution of strain TYQ2 was centrifuged at 5,000 *g* for 10 min to obtain cells, then washed with 10 mM phosphate buffered saline (PBS, pH 7.4) and centrifuged at 5,000 *g* for 5 min. Washing and centrifugation operations were repeated three times as above. Then, bacterial cells were resuspended, where the copper grid coated with carbon film was immersed for 20 min. Finally, the ready-made copper grid was dried at indoor temperature for 20 min, and then observed under TEM (Buchan et al., 2014).

### Studies on Physiological and Biochemical Traits of Strain TYQ2

The ranges of temperature and pH for the growth of strain TYQ2 were tested and indicated by the absorbance of OD_600_ value in the 1/10 2216E liquid medium (0.5 g tryptone and 0.1 g yeast extract in 1-liter filtered seawater). The range of NaCl concentration was tested in the modified 1/10 2216E liquid medium. Growth tests were performed at different temperatures (4, 10, 16, 28, 30, 37, and 40°C). Sodium chloride tolerance was detected in the modified medium (0.5 g tryptone and 0.1 g yeast extract dissolved in 1-liter distilled water) supplemented with 0–10% (w/v) NaCl (1.0% intervals). The pH range was tested from 2.0 to 12.0 (increments of 1 pH unit). Some of physiological characteristics were determined by using the API 20NE (Biomerieux, Lyon, France) tests. To test the utilization of various electron donors and energy sources by strain TYQ2, the 1/10 2216E liquid medium added without or with single substrates (including glucose, sucrose, fructose, lactose, maltose, xylose, rhamnose, xylan, mannose, arabinose, inositol, glycerol, sodium pyruvate, sodium acetate, sodium citrate, sodium propionate, formate, salicylic acid, succinate, mannitol, cellulose, starch, glycine, trehalose, ethanol, polyethylene glycol, D-sorbitol) at 10 mM was used to test the growth of strain TYQ2. All the culture conditions were incubated at 28°C for 5 days. To study the effects of various sulfur sources on the growth of strain TYQ2, the growth of bacterial cells in the 1/10 2216E medium supplemented without or with 2% DMSO, 20 mM Na_2_S_2_O_3_, 5 mM Na_2_SO_3_, or 100 mM Na_2_SO_4_ were measured by OD_600_ value as described above. Three biological replicates were performed for both control and experimental groups.

### Genomic Sequencing and Analysis of Strain TYQ2

To determine the whole genome sequences, genomic DNA was extracted from 5 days’ bacterial culture of the strain TYQ2. The DNA library was constructed by Nanopore PromethION platform and Illumina NovaSeq platform at the Beijing Novogene Bioinformatics Technology Co., Ltd. Firstly, large DNA fragments were recovered by Blue Pippin automatic nucleic acid fragment recovery system, and then repaired. Next, barcode was added by PCR-free method of EXP-NBD104 kit from Oxford Nanopore Technologies Company. The fragments’ size was detected by AATI automatic capillary electrophoresis instrument to get the samples isomolarly mixed. Afterward, the SQK-LSK109 connection kit was used to connect the adapter and the library was preliminarily constructed. Then, sequencing libraries were generated by using NEBNext^®^ Ultra™ DNA Library Prep Kit for Illumina (NEB, Ipswich, MA, United States). PE150 data and Nanopore data were combined to assemble by using Unicycler.

### Phylogenetic Analysis

Phylogenetic tree based on 16S rRNA gene sequences of strain TYQ2 and some other related taxa was constructed by the maximum likelihood method. Briefly, the 16S rRNA gene (1,511 bp) of strain TYQ2 was obtained from its assembled complete genome (accession number CP076053.1). Other 16S rRNA gene sequences were obtained from the type strains in the NCBI GenBank database.^1^ The multiple sequence alignment and sequences’ trim were performed by the MEGA version 5.0 software. Finally, the phylogenetic tree was completed by using W-IQ-TREE web server^2^ (Trifinopoulos et al., 2016) with TIM3 + F + I + G4 model. The edition was performed by the website tool: Interactive Tree of Life^3^ (Letunic and Bork, 2019).

### Growth Assays and Transcriptomic Analysis

To detect the effect of taurine on the growth, strain TYQ2 was cultured in the modified 1/10 2216E medium supplemented with different concentrations of taurine (0, 10, 20, 30, 40, 50 mM), and OD_600_ values were checked on the third day with a microplate reader (Infinite M1000 Pro; Tecan, Mannedorf, Switzerland). Since the experimental group had the most obvious effect of promoting growth on the third day, 3 days’ cell cultures of strain TYQ2 in the above 1/10 2216E medium supplemented with (40 mM) or without taurine were respectively collected by centrifugation at 5000 *g*, 4°C, 10 min for transcriptomic analysis. In addition, as the same as above, strain TYQ2 was cultured in the modified 1/10 2216E medium supplemented without or with 10 g/L polyvinyl alcohol, respectively. Daily growth was recorded by OD_600_ values and plotted as a growth curve. Four days’ culture of the two conditions was used for transcriptomic analysis, for the same reasons as before. Three parallel replicates were set for all the growth tests above.

According to the manufacturer (Novogene, Beijing, China), the brief procedures of transcriptomic analyses were as follows. First, total RNAs of the strain TYQ2 were extracted and rRNA was removed by using probes. Purified mRNA was reversely transcribed into cDNA, and then cDNA was used to prepare the library. After accurate assessment of the library quality and cluster generation, the library preparations were sequenced on an Illumina Novaseq platform. Then, based on the high-quality clean data and genome website, Bowtie2 (Langmead and Salzberg, 2012) was used to both build index of reference genome and align clean reads to reference genome. Gene expression level was estimated by FPKM (Trapnell et al., 2009). Differential expression analysis of two conditions/groups was performed using the DESeq R package (Anders and Huber, 2010). GO and KEGG enrichment analyses of differentially expressed genes were tested by the GOseq R package (Young et al., 2010) and KOBAS software respectively.

## Results and Discussion

### Cultivation and Phylogeny of a Deep-Sea Actinobacterium

**FIGURE 1:**
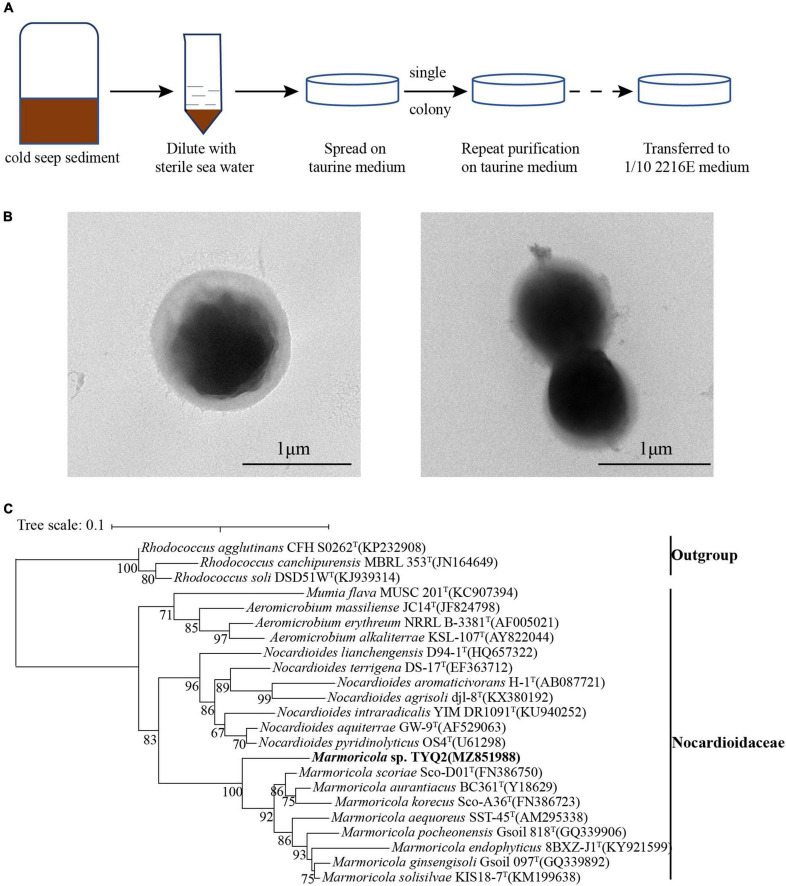
Cultivation, morphology, and phylogeny of *Marmoricola* sp. TYQ2 isolated from the deep-sea cold seep. **(A)** Diagram of enrichment and cultivation of strain TYQ2 by using a taurine supplemented medium. **(B)** Observation of the cell morphology of strain TYQ2 through the transmission electron microscopy. Bar is 1 μm. **(C)** Maximum likelihood 16S rRNA gene phylogenetic tree showing the position of strain TYQ2 as well as the family *Nocardioidaceae* within the *Actinobacteria* phylum. The accession number of each 16S rRNA gene is shown in the parentheses after corresponding strain name. The numbers by the side of branch node indicated statistical support for bootstrap values, respectively. Scale bar, 0.1 substitutions per nucleotide position.

Given that taurine is an important nutrient source for marine prokaryotes (Clifford et al., 2019), we developed an approach for enrichment of taurine-utilization microorganisms by using a basal medium supplemented with taurine (Figure 1A). With that, deep-sea cold seep samples diluted with sterile seawater were spread on plates containing a basal medium supplemented with appropriate amount of taurine and incubated at 28°C. After 2 weeks, we only observed a few white colonies on the plate. Through successive generations of purification and 16S rRNA gene sequencing confirmation, a pure bacterial strain named TYQ2 was obtained (Figure 1A). It is observed under TEM as shown in Figure 1B. According to the 16S rRNA sequence analysis, strain TYQ2 was identified as a member of the phylum *Actinobacteria*.

To gain a deeper insight into strain TYQ2, its entire genome was sequenced and analyzed (Supplementary Figure 1). The whole genome size of strain TYQ2 was 3,600,424 bp, and the DNA G + C content was 71.99%. The annotation of the genome of strain TYQ2 revealed that it consisted of 3,440 predicted genes, including 52 RNA genes (6 rRNA genes, 46 tRNA genes) (Supplementary Table 1).

To further clarify the taxonomic status of the strain TYQ2, we performed the phylogenetic analysis with 16S rRNA genes of the cultured type strains of *Nocardiaceae* and other actinomycete groups as outgroup by using the maximum likelihood method (Figure 1C). Based on the 16S rRNA sequence of strain TYQ2, the sequence similarity calculation using the NCBI server showed that strain TYQ2 was closely related to the *Marmoricola aurantiacus* strain BC 361^T^ (95.94% similarity), which was a type strain of the genus *Marmoricola* isolated from a marble statue (Urzl et al., 2000).

It is noting that we also obtained some species belonging to the phylum *Actinobacteria* from the deep-sea cold seep sediments through the same enrichment and cultivation method, suggesting the actinomycete group has a universal trait of metabolizing taurine. Since taurine was an organic sulfonate widely distributing in diverse marine environments (Clifford et al., 2017, 2019), it would be an available way to cultivate other uncultured microorganisms in the future.

### Physiological and Biochemical Characteristics

The basic physiological and chemical characteristics of strain TYQ2 were summarized in the Supplementary Table 2. In detail, the colony of strain TYQ2 was round, smooth, convex, and light yellow in color. Transmission electron microscopic (TEM) observation showed that strain TYQ2 possessed a regular-shaped spherical cell (about 0.4–1.0 μm in size) without flagellum (Figure 1B). Gram-reaction-positive, aerobic, non-motile. Growth was detected at temperatures between 10°C and 37°C, but no growth was detected at temperatures below 10°C or above 37°C, and the optimal growth temperature was 28°C. Strain TYQ2 could grow at a pH value of 5.0–10.0, and the optimum pH was 7.0. Growth was detected between 0 and 8.0% NaCl concentration, and the optimum salinity was 4%. Oxidase was negative. In API 20NE tests, positive for aesculin hydrolysis, acid production from glucose and arginine dihydrolase, urease and gelatinase activities, but negative for nitrate and nitrite reduction and indole production. P-Nitro-β-D galactose, glucose, arabinose, mannose, N-acetyl-glucosamine, maltose, gluconate, malic acid, citric acid, and phenylacetic acid were utilized, but mannitol, capric acid, and adipic acid were not. In supplemental growth assays, growth of strain TYQ2 was stimulated by adding glucose, sucrose, fructose, maltose, xylose, rhamnose, xylan, mannose, arabinose, glycerol, sodium pyruvate, sodium acetate, sodium citrate, sodium propionate, cellulose, starch, trehalose or D-sorbitol as a carbon source or electron donor. But adding lactose, inositol, formate, salicylic acid, succinate, mannitol, glycine, ethanol, or polyethylene glycol could not promote growth. In addition, the effects of different sulfur sources on the growth of strain TYQ2 were observed, including 2% DMSO, 20 mM Na_2_S_2_O_3_, 5 mM Na_2_SO_3_, and 100 mM Na_2_SO_4_ (Supplementary Figure 2A). It was found that 2% DMSO, 20 mM Na_2_S_2_O_3_, and 5 mM Na_2_SO_3_ inhibited the growth of strain TYQ2. 100 mM Na_2_SO_4_ could promote the growth of strain TYQ2. The strain *Marmoricola* sp. TYQ2 was isolated from the deep-sea cold seep sediments in the South China Sea.

### Strain TYQ2 Possessed a Prominent Capability of Utilizing Taurine

**FIGURE 2:**
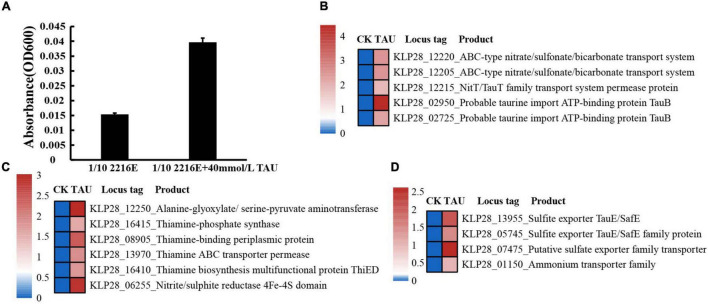
Strain TYQ2 effectively utilizes taurine. **(A)** Growth assays of strain TYQ2 in the 1/10 2216E medium supplemented either without or with 40 mM taurine. **(B)** A heat map based on transcriptomics analysis showing the up-regulated genes associated with taurine and sulfonate transport. **(C)** A heat map based on transcriptomics analysis showing the up-regulated genes associated with the metabolism of taurine. **(D)** A heat map based on transcriptomics showing the up-regulated genes involved in metabolites export.

Based on our strategy of using taurine as the sole carbon source for isolation and cultivation of strain TYQ2, we speculate that this strain should be able to utilize taurine. We thus checked the growth of strain TYQ2 in a basal 1/10 2216E medium supplemented with taurine at a final concentration of 0, 10, 20, 30, 40, or 50 mM (Supplementary Figure 2B). The results showed that the growth of strain TYQ2 was promoted along with the increase of the concentration of taurine. Especially at the concentration of 40 mM, the growth rate was increased by nearly 3 times compared with the control group (Figure 2A). To further disclose the details of taurine utilization by strain TYQ2, we performed a transcriptome analysis of strain TYQ2 grown in 1/10 2216E medium supplemented without or with taurine (at a final concentration of 40 mM). Indeed, when 40 mM taurine was added to the medium, the expressions of many genes encoding key factors associated with taurine transport (Figure 2B), taurine degradation (Figure 2C), and metabolites export (Figure 2D) were evidently up-regulated. Similar to the reported metabolic process of taurine (Cook and Denger, 2006; Nishikawa et al., 2018), taurine or sulfonate transporter could transport taurine into the cell (Figure 2B); transamination enzyme and thiamine-phosphate synthase then promoted the destruction of taurine structure (Figure 2C); finally, the metabolites were exported by sulfite exporters and ammonium export genes (Figure 2D). The destruction of taurine’s structure was accompanied by the production of acetyl-CoA, thereby entering the tricarboxylic acid cycle (Cook and Denger, 2006; Nishikawa et al., 2018).

**FIGURE 3:**
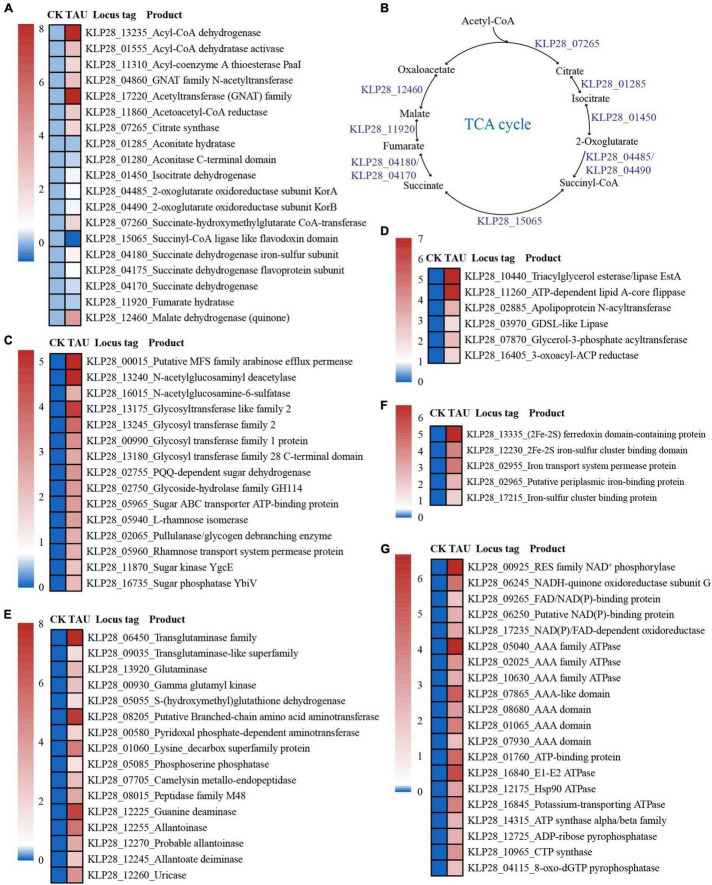
Transcriptomic analysis of essential metabolic pathways for energy production in strain TYQ2 that cultured in the medium supplemented with taurine. **(A)** A heat map based on transcriptomics showing the up-regulated genes involved in acetyl-CoA synthesis and metabolism and the tricarboxylic acid cycle. **(B)** The diagram of the tricarboxylic acid cycle of strain TYQ2. The gene numbers are the same to those shown in panel **(A)**. **(C)** A heat map based on transcriptomics showing all the up-regulated genes involved in sugar transport and metabolism. **(D)** A heat map based on transcriptomics showing all up-regulated genes related to lipid metabolism. **(E)** Transcriptomics-based heat map showing all up-regulated genes related to amino acid and nucleotide metabolism. **(F)** A heat map based on transcriptomics showing all up-regulated genes encoding iron-sulfur proteins. **(G)** A heat map based on transcriptomics showing the up-regulation of all genes related to NAD(P)/FAD/NADH oxidoreductase and ATPase.

Consistently, the expressions of genes encoding key proteins associated with the acetyl-CoA utilization and tricarboxylic acid cycle (including citrate synthase, aconitate hydratase, isocitrate dehydrogenase, 2-oxoglutarate oxidoreductase, succinyl-CoA ligase, succinate dehydrogenase, fumarate hydratase, and malate dehydrogenase) were mostly up-regulated (Figures 3A,B). In addition, the expression levels of genes encoding essential proteins closely related to saccharide metabolism (Figure 3C), lipid metabolism (Figure 3D), amino acid and nucleotide metabolism (Figure 3E), iron-sulfur proteins (Figure 3F) were also significantly up-regulated. Consequently, the expression levels of many proteins related to ATP production [including ATP synthase, ATPase, FAD/NAD(P)-binding protein] were also significantly up-regulated (Figure 3G). The above results strongly suggested that the metabolization and utilization of taurine could effectively promote the energy production, thereby facilitating the growth of strain TYQ2.

Taurine was an amino acid-like compound containing +5 oxidation state sulfur atom, belonging to the naturally occurring organic sulfonates (Cook and Denger, 2002). Taurine was found in a variety of organisms in the marine environment, including algae, oysters, copepods, diatoms, and various marine metazoans. It acts as an osmotic protective agent or performs other important physiological functions like amino acid metabolism in the organisms, and can be released into the ocean through different pathways (Jackson et al., 1992; Pierce et al., 1992; Clifford et al., 2017, 2019). Previous research reported that organic sulfonates accounted for 20–40% of the total organic sulfur in marine sediments (Vairavamurthy et al., 1994), and taurine acted as one of important components. Diverse bacteria have the ability to degrade taurine, and the degradation products (sulfate, sulfite, sulfide, thiosulfate, low-molecular-weight organic sulfonates, ammonium, alanine, etc.) may also be vital elements and energy sources for other metabolic processes (Lie et al., 1999; Visscher et al., 1999; Cook and Denger, 2006; Krejcik et al., 2008). Due to zooplankton and other metazoans releasing taurine in some nutrient-limited environments, taurine was particularly important for meso- and bathypelagic prokaryotes (Clifford et al., 2019). *Actinobacteria* members widely distributed in the ocean, and they were believed to be essential players driving the biogeographic cycle process of marine environments (Mincer et al., 2002; Miralles et al., 2021; Rathore et al., 2021). Metabolism of taurine by *Actinobacteria* may promote deep-sea elements and nutrient cycling processes. However, until now, there is rarely information on the metabolism of taurine by marine *Actinobacteria*, especially the deep ocean.

In the present study, we enrich and culture a deep-sea actinomycete strain TYQ2 with taurine as the sole carbon source, demonstrate its prominent capability toward the degradation and utilization of taurine, and discover the preference for taurine of *Actinobacteria*. Based on the current research, the process of bacterial degradation of taurine has been reported mostly through the same metabolic pathway. The biotransformation of taurine in bacteria usually requires specialized sulfonate transporters to be transported into the cell, and the intracellular taurine is transformed to alanine, HSO_3_^–^, and acetyl phosphate under the combined action of transamination enzyme and thiamine-phosphate synthase. Bisulfite or sulfate ions participates in intracellular sulfur metabolism or be excreted through transporters. Alanine participates in intracellular amino acid metabolism, and acetyl phosphate can generate acetyl-CoA to participate in intracellular tricarboxylic acid cycle and energy metabolism (Cook and Denger, 2006; Nishikawa et al., 2018). In conclusion, the entire process of strain TYQ2’s taurine metabolism involves energy production and the flow of carbon, nitrogen, and sulfur elements.

### Strain TYQ2 Possessed a Prominent Capability of Utilizing Polyvinyl Alcohol

**FIGURE 4:**
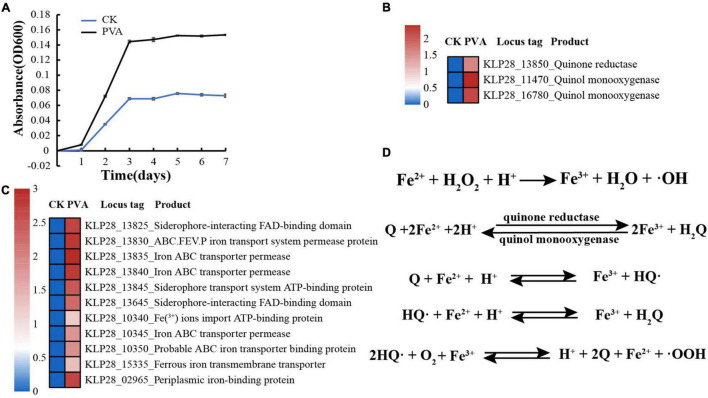
Strain TYQ2 effectively utilizes PVA. **(A)** Growth assays of strain TYQ2 in the 1/10 2216E medium supplemented either without or with 10 g/L PVA. **(B)** A heat map based on transcriptomics analysis showing the up-regulation of genes encoding the quinone reductase and quinol monooxygenase. **(C)** A heat map based on transcriptomics analysis showing the up-regulated genes related to iron/ferrous iron transmembrane transporters and relevant FAD/ATP-binding proteins. **(D)** The proposed Fenton reaction driven by the redox process of quinone and quinol. Q, quinone; H_2_Q, quinol; HQ, semiquinone; OOH, perhydroxyl radical.

**FIGURE 5:**
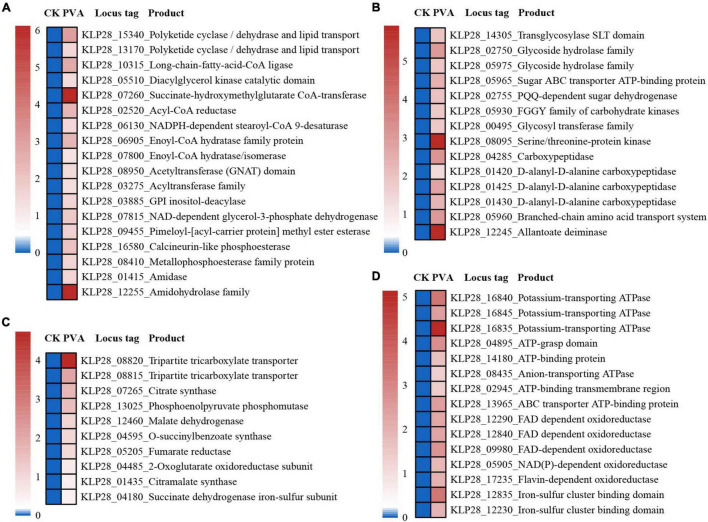
Transcriptomic analysis of essential metabolic pathways for energy production in strain TYQ2 that cultured in the medium supplemented with PVA. **(A)** A heat map based on transcriptomics analysis showing the up-regulated genes related to lipid metabolism. **(B)** A heat map based on transcriptomics showing all the up-regulated genes associated with sugar/amino acid/nucleotide metabolism. **(C)** A heat map based on transcriptomics analysis showing all up-regulated genes related to tricarboxylic acid cycle. **(D)** A heat map based on transcriptomics analysis showing all up-regulated genes encoding proteins associated with ATP generation.

When we investigated the proper metabolized substrates of strain TYQ2, we found that PVA could significantly accelerate its growth (2∼3 times) (Figure 4A). To explore the degradation and utilization mechanisms of PVA by strain TYQ2, we performed the transcriptomic analysis of strain TYQ2 cultured in the medium supplemented with PVA. According to previous reports (Kerem et al., 1999; Larking et al., 1999; Jensen et al., 2001; Baldrian and Valaskova, 2008; Kawai and Hu, 2009), some microorganisms could use the redox cycle of extracellular quinol and quinone to reduce iron ions to ferrous ions and produce H_2_O_2_, which enabled microorganisms to degrade various organic compounds. Based on our transcriptomic results, the expressions of genes encoding quinone reductase and quinol monooxygenase were significantly up-regulated (Figure 4B). Additionally, the expressions of many genes encoding Fe^2+^/Fe^3+^ transmembrane transporters and relevant FAD/ATP-binding proteins were also markedly up-regulated (Figure 4C). Therefore, we speculate that strain TYQ2 could degrade PVA through the same pathway shown in Figure 4D. In fact, when the conversion process above happened, quinol (H_2_Q) was oxidated by Fe^3+^ to semiquinone radicals (HQ⋅), the semiquinone could reduce O_2_ to produce ⋅OOH and quinone. Strongly oxidizing peroxides like H_2_O_2_ originated from perhydroxyl radicals (⋅OOH). Meanwhile, Fe^3+^ was reduced by semiquinone to Fe^2+^ and quinone could be reduced back to quinol again by ferrous ions. In this way, the whole reaction process was carried out cyclically, a complete Fenton system and lots of perhydroxyl radicals were continuously generated (Kerem et al., 1999; Larking et al., 1999; Jensen et al., 2001; Baldrian and Valaskova, 2008; Kawai and Hu, 2009). The whole process was shown the same as Figure 4D and these strongly oxidizing perhydroxyl radicals could destroy the structure of polyvinyl alcohol effectively. These indicated that the strain TYQ2 could also reduce iron ions to ferrous ions through the extracellular quinone reduction and quinol oxidation process and generate perhydroxyl radicals. Then, the cyclic Fenton reaction got achieved and the structure of polyvinyl alcohol was destroyed to be utilized. On the other hand, according to previous reports, esterase and lipid metabolism-related genes could promote the metabolism of low-molecular-weight PVA components (Sakai et al., 1998; Chiellini et al., 2003). Consistent with growth promotion of strain TYQ2 by the supplement of PVA in the medium, the expressions of many genes related to lipid metabolism (Figure 5A), sugar/amino acid/nucleotide metabolism (Figure 5B), tricarboxylic acid cycle (Figure 5C), and ATP generation (Figure 5D) were significantly up-regulated, suggesting strain TYQ2 not only degraded PVA but also utilized corresponding degradation products as an energy source.

Polyvinyl alcohol was currently the world’s highest output water-soluble synthetic polymer, and its earlier annual output could reach 650,000 tons (Tokiwa et al., 2001). As the global production and consumption of PVA were expected to continuously increase, the content of PVA flowing into the environment accordingly showed an increasing trend (Kawai and Hu, 2009). As a potential plastics contaminant, PVA has an adverse effect on the ecological environment, especially the marine environment (Chiellini et al., 2003). Some microorganisms like strain TYQ2 could utilize PVA, which may alleviate the contamination (Suzuki et al., 2021). On the other hand, it is noting that plastics are predominantly carbon at the elemental level (Stubbins et al., 2021). Accumulated studies reveal the quantities of plastics and corresponding degraded intermediates present in some ecosystems rival the quantity of natural organic carbon and impact the carbon cycling of the world (Stubbins et al., 2021), suggesting that geochemists and ecologists should now consider plastics in their analyses. In the same way, combined with the metabolic utilization of PVA by strain TYQ2, this metabolic process involves the utilization and flow of carbon elements.

**FIGURE 6:**
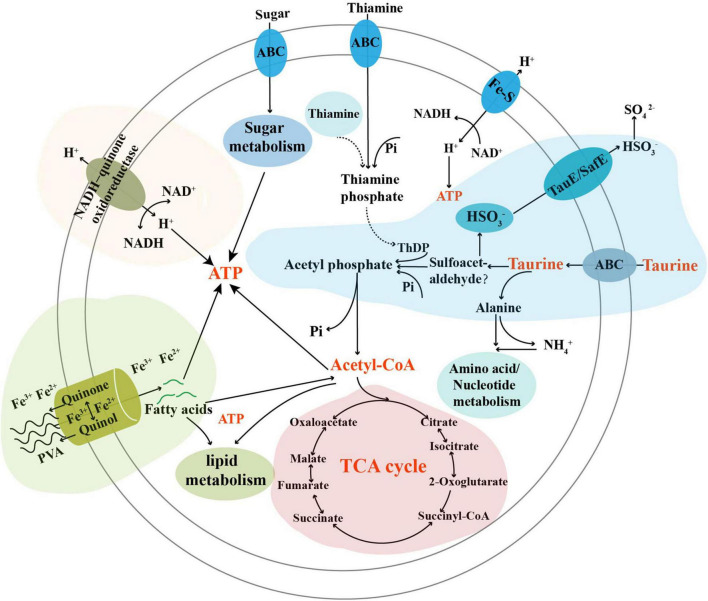
Diagram of a proposed model describing degradation and utilization of taurine and PVA by *Marmoricola* sp. TYQ2. In this model, the metabolization of taurine and PVA as well as the contribution to energy production was highlighted. ThDP, thiamine diphosphate; Pi, phosphate group.

Combining the data on the metabolization of taurine and PVA, as well as the growth promotion of strain TYQ2, we proposed a model representing the central metabolism of strain TYQ2 (Figure 6). In this model, taurine was transported into the cells by special transporters, and then was degraded thereby entering into the sulfur cycle; the degraded products might be utilized by strain TYQ2 and transformed to ATP through different metabolic pathways, thereby promoting bacterial growth. On the other hand, PVA might be degraded with the involvement of the redox cycle of extracellular quinol and quinone and the reduction of iron to ferrous; the degradation products could also be utilized by strain TYQ2, thereby promoting the energy production and supporting bacterial growth. In addition, amplicon sequencing analysis of our deep-sea sediment samples revealed the distribution and abundance of *Actinobacteria* here (Supplementary Figure 3). Combined with a lot of previous reports, *Actinobacteria* are widely distributed in marine and deep-sea environments, whether nearshore or pelagic, deep-sea troughs or trenches, ordinary seas or extreme deep-sea environments such as cold seeps and hydrothermal vents (Colquhoun et al., 1998a,b; Bull et al., 2005; Prieto-Davó et al., 2013; Li and Wang, 2014; Mamaeva et al., 2016; Han et al., 2018). Therefore, the members of *Actinobacteria* may play a key role in the degradation and utilization of endogenous or exogenous organic matter, thereby contributing to the nutrient and elements cycling of the deep biosphere. In conclusion, this study explored, revealed and discussed the outstanding metabolic capacity and complete metabolic process of taurine and PVA by the actinomycete strain TYQ2, which involved energy production and flow of several types of elements, and the potential value of deep-sea *Actinobacteria* was prospected.

## Data Availability Statement

The 16S rRNA gene sequence and complete genome sequence of *Marmoricola* sp. TYQ2 have been, respectively, deposited in the GenBank database under the accession numbers MZ851988.1 and CP076053.1. The original sequencing reads for transcriptomic analysis have been deposited to the NCBI Short Read Archive. The accessions were, respectively, SAMN21031663, SAMN21031664, SAMN21031665, and SAMN21031666. The raw amplicon sequencing data have been deposited to the NCBI Short Read Archive under the accession number PRJNA675395. Strain TYQ2 has been preserved in the China General Microbiological Culture Collection Center, Beijing, China (accession number: CGMCC1.19148).

## Author Contributions

YT and CS conceived and designed the study and led the writing of the manuscript. YT conducted most of the experiments. YS collected the samples from the deep-sea cold seep. RZ helped to isolate the bacterium. RL helped to perform the OTU analysis. All authors reviewed the manuscript and approved the submitted version.

## Conflict of Interest

The authors declare that the research was conducted in the absence of any commercial or financial relationships that could be construed as a potential conflict of interest.

## Publisher’s Note

All claims expressed in this article are solely those of the authors and do not necessarily represent those of their affiliated organizations, or those of the publisher, the editors and the reviewers. Any product that may be evaluated in this article, or claim that may be made by its manufacturer, is not guaranteed or endorsed by the publisher.

## Footnotes

1
https://www.ncbi.nlm.nih.gov/

2
http://iqtree.cibiv.univie.ac.at/

3
https://itol.embl.de/
