# Supplementary material for: Characterization of a Deep-Sea Actinobacterium Strain Uncovers Its Prominent Capability of Utilizing Taurine and Polyvinyl Alcohol
Source: Front Microbiol. 2022 May 23;13:868728. doi: 10.3389/fmicb.2022.868728 (PMC9169050; doi:10.3389/fmicb.2022.868728)
Supplement: Supplementary file 1 [file Data_Sheet_1.pdf]

Supplementary Material

1. Supplementary Methods

Quantitative Amplicon Analysis

To better understand the distribution of different types of bacteria and the abundance of *Actinobacteria* in deep-sea cold seep sediments, we collected deep-sea samples by *RV KEXUE* from a typical cold seep in the South China Sea. Then, some sediment samples (including RPC, ZC1, ZC2, ZC4, ZC5), were used for amplicon sequencing of 16S rRNA gene (Novogene, China) （Supplementary Table 3）. The sampling depth were 0-10, 30-50, 90-110, 210-230 and 230-250 cm, respectively. The specific steps were as following: firstly, the total DNAs of the samples were respectively extracted by the CTAB/SDS method (Murray and Thompson, 1980), and diluted with sterile water to 1 ng/μL as a PCR template. Specific primers (341F：5’-CCTAYGGGRBGCASCAG and 806R：5’-GGACTACNNGGGTATCTAAT) were used to amplify 16S rRNA genes in different regions (16S V3/V4), and these PCR products were purified with Qiagen Gel Extraction Kit (Qiagen, Germany) to build the library. After quality evaluation, the library was sequenced on the Illumina NovaSeq platform to generate 250 bp paired-end reads. FLASH (V1.2.7, http://ccb.jhu.edu/software/FLASH/) (Magoc and Salzberg, 2011) was used to merge paired-end reads and generate raw tags. High-quality tag sequences were obtained from quality filtered original tags by the control process of QIIME (V1.9.1, http://qiime.org/scripts/split_libraries_fastq.html) (Bokulich et al., 2013). These tag sequences were aligned with the reference database. UCHIME algorithm (http://www.drive5.com/usearch/manual/uchime_algo.html) (Edgar et al., 2011) was used to detect the chimeric sequences (Haas et al., 2011). After removing chimeric sequences, sequence analysis was performed by using Uparse software (Uparse v7.0.1001, http://drive5.com/uparse/) (Edgar, 2013). For each representative sequence, the Silva database (http://www.arb-silva.de/) (Quast et al., 2013) was used to annotate the taxonomic information based on the Mothur algorithm.

References

Bokulich, N.A., Subramanian, S., Faith, J.J., Gevers, D., Gordon, J.I., Knight, R., et al. (2013). Quality-filtering vastly improves diversity estimates from illumina amplicon sequencing. *Nat. Methods* 10, 57-U11. doi: 10.1038/Nmeth.2276.

Edgar, R.C. (2013). UPARSE: highly accurate OTU sequences from microbial amplicon reads. *Nat. Methods* 10, 996-998. doi: 10.1038/Nmeth.2604.

Edgar, R.C., Haas, B.J., Clemente, J.C., Quince, C., and Knight, R. (2011). UCHIME improves sensitivity and speed of chimera detection. *Bioinformatics* 27, 2194-2200. doi: 10.1093/bioinformatics/btr381.

Haas, B.J., Gevers, D., Earl, A.M., Feldgarden, M., Ward, D.V., Giannoukos, G., et al. (2011). Chimeric 16S rRNA sequence formation and detection in Sanger and 454-pyrosequenced PCR amplicons. *Genome Res.* 21, 494-504. doi: 10.1101/gr.112730.110.

Magoc, T., and Salzberg, S.L. (2011). FLASH: fast length adjustment of short reads to improve genome assemblies. *Bioinformatics* 27, 2957-2963. doi: 10.1093/bioinformatics/btr507.

Murray, M.G., and Thompson, W.F. (1980). Rapid isolation of high molecular-weight plant DNA. *Nucleic Acids Res.* 8, 4321-4325. doi: 10.1093/nar/8.19.4321.

Quast, C., Pruesse, E., Yilmaz, P., Gerken, J., Schweer, T., Yarza, P., et al. (2013). The SILVA ribosomal RNA gene database project: improved data processing and web-based tools. *Nucleic Acids Res.* 41, D590-D596. doi: 10.1093/nar/gks1219.

2. Supplementary Figures and Tables

2.1 Supplementary Figures

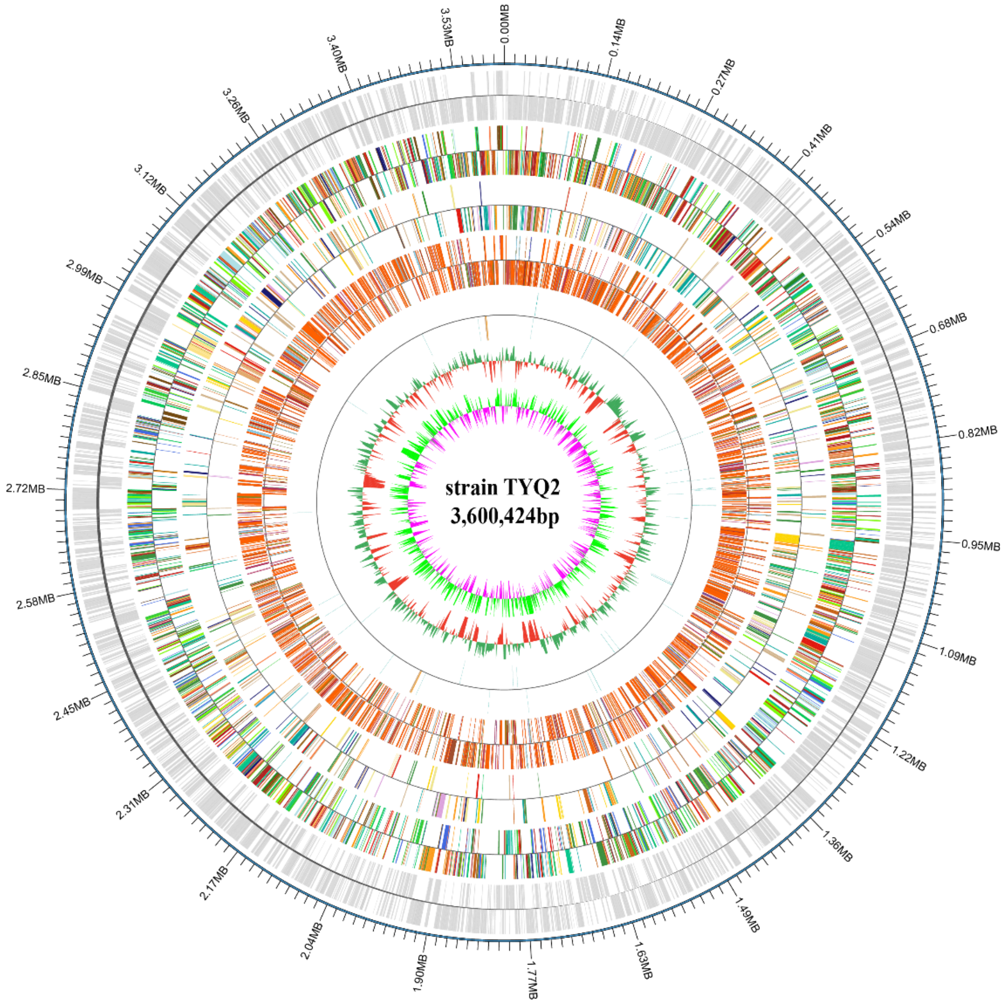

Supplementary Figure 1. Circular diagram of the genome of *Marmoricola* sp. TYQ2. The outermost circle was the position coordinates of the genome sequence. From the outside to the inside: the coding genes, gene function annotation results, ncRNA, and genome GC content. The inward red part: the GC content of this region was lower than the average GC content of the whole genome; the outward green part: the GC content of this region was higher than the average GC content of the whole genome. The higher the peak value, the greater the difference from the average GC content. Genome GC skew value: the inward pink part: the content of G in this region was lower than the content of C; the light green part outside: the content of G in this region was higher than the content of C.

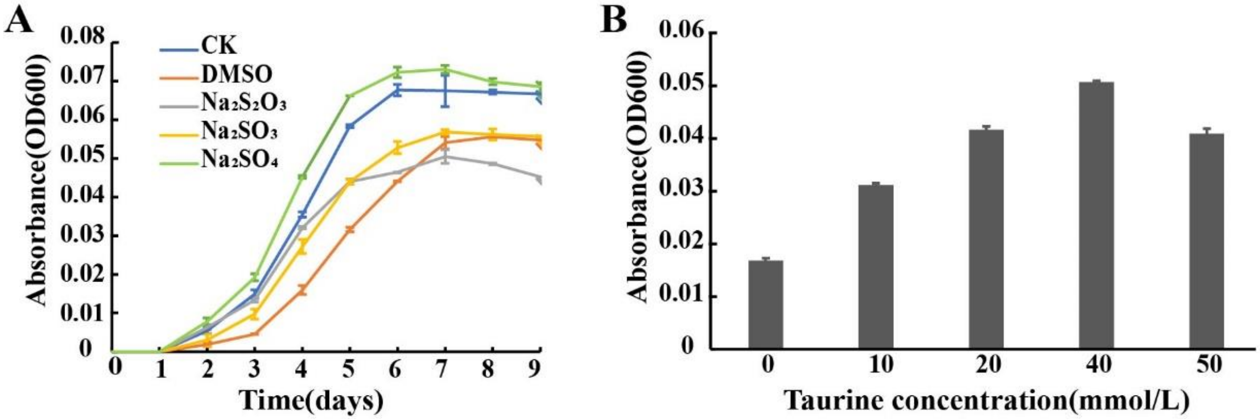

Supplementary Figure 2. Effects of different sulfur sources and different concentrations of taurine on the growth of strain TYQ2. (A) The growth curves of strain TYQ2 in basal 1/10 2216E medium (CK) and basal medium supplemented with 2% DMSO, 20 mM $Na_2S_2O_3$, 5 mM Na_2SO_3 and 100 mM Na_2SO_4, respectively. (B) The growth of strain TYQ2 in basal 1/10 2216E medium supplemented with different concentrations of taurine (0, 10, 20, 30, 40 or 50 mM).

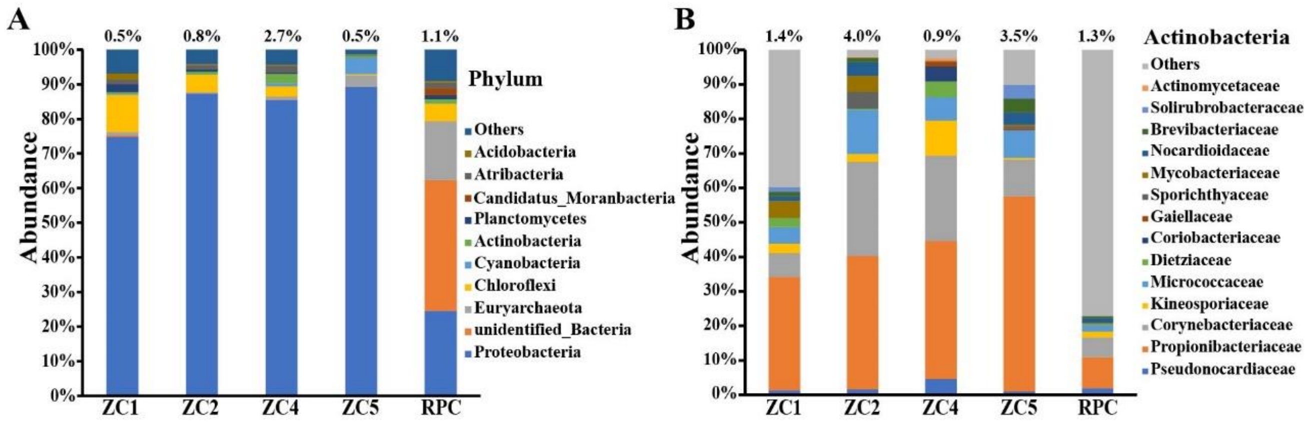

Supplementary Figure 3. Quantitative analysis of the distribution of different species of bacteria and the abundance of *Actinobacteria* and *Nocardioidaceae* in deep-sea cold seeps. (A) Quantitative analysis of the distribution of different bacterial species and abundance of *Actinobacteria* members in different layers of deep-sea sediments (including ZC1, ZC2, ZC4, ZC5, and RPC) by 16S rRNA gene amplicon sequencing. The relative abundances of operational taxa (OTUs) representing different bacteria are shown at the phylum level. (B) Quantification of the abundance of *Nocardioidaceae* members in the phylum *Actinobacteria* based on the amplicon sequencing.

2.2 Supplementary Tables

Supplementary Table 1. Genomic features of *Marmoricola* sp. TYQ2

**Genomic features**	**TYQ2**
Gene Bank ID	CP076053.1
Genome size (bp)	3,600,424
G+C content (%)	71.99
Chromosomes	1
No. of genes	3440
No. of rRNAs	2, 2, 2 (5S, 16S, 23S)
No. of tRNAs	46
Completeness (%)	100

Supplementary Table 2. Physiological and biochemical characteristics of *Marmoricola* sp. TYQ2

Characteristic	TYQ2
Cell length (μm)	0.4-1.0 (μm)
Cell shape	Coccus
Gram stain	Positive
Sporulation	No-sporulation
Aerobic or anaerobic	Aerobic
Temperature range for growth [Optimum] (°C)	10-37 (28)
pH range for growth [Optimum]	5-10 (7)
NaCl range for growth [Optimum] (%)	0-8(4)
Utilization as an electron donor or an added energy source:	
glucose	+
sucrose	+
fructose	+
lactose	-
maltose	+
xylose	+
rhamnose	+
xylan	+
mannose	+
arabinose	+
inositol	-
glycerol	+
sodium pyruvate	+
sodium acetate	+
sodium citrate	+
sodium propionate	+
formate	-
salicylic acid	-
succinate	-
mannitol	-
cellulose	+
starch	+
glycine	-
trehalose	+
ethanol	-
polyethylene glycol	-
D-sorbitol	+

Supplementary Table 3. Sample location information

Sample location	Cold seep
Site name	Taixinan
Sample type	deep-sea sediments
Latitude and longitude	119°17′05.3940″E<br>22°06′58.7264″N
Depth under the surface of ocean (m)	1,173
Depth under the surface of sediment (cm)	0~250
